# Antioxidant and Antibacterial Capacities of *Origanum vulgare* L. Essential Oil from the Arid Andean Region of Chile and its Chemical Characterization by GC-MS

**DOI:** 10.3390/metabo10100414

**Published:** 2020-10-16

**Authors:** Mario J. Simirgiotis, Daniel Burton, Felipe Parra, Jéssica López, Patricio Muñoz, Hugo Escobar, Claudio Parra

**Affiliations:** 1 Instituto de Farmacia, Facultad de Ciencias, Universidad Austral de Chile, Valdivia 5110566, Chile; mario.simirgiotis@uach.cl; 2 Laboratorio de Productos Naturales y Química Médica, Facultad de Ciencias Agronómicas, Universidad de Tarapacá, Av. General Velásquez 1775, Arica 1000000, Chile; burtonvillalobos@gmail.com (D.B.); filyparramontes@hotmail.com (F.P.); pmunozt@ucdavis.edu (P.M.); 3 Escuela de Alimentos, Pontificia Universidad Católica de Valparaíso, Valparaíso 2360100, Chile; j.lopez.pasten@gmail.com; 4 University of California Davis Chile Life Sciences Innovation Center, Santiago 7511303, Chile

**Keywords:** antibacterial activity, antioxidant activity, essential oil, GC-MS, oregano, Putre

## Abstract

This study aimed to characterize the in vitro antioxidant and antibacterial properties of oregano (*Origanum vulgare*) essential oil, as well as its chemical composition. To our best knowledge, there are few studies on oregano grown in the arid Andes region, but none on the metabolites produced and their bioactivity. This work identified fifty metabolites by Gas Chromatography–Mass Spectrometry (GC-MS)—monoterpene hydrocarbons, oxygenated monoterpenes, phenolic monoterpenes, sesquiterpene hydrocarbons, and oxygenated sesquiterpenes—present in the essential oil of oregano collected in the Atacama Desert. The main components of essential oregano oil were thymol (15.9%), *Z*-sabinene hydrate (13.4%), γ-terpinene (10.6%), *p*-cymene (8.6%), linalyl acetate (7.2%), sabinene (6.5%), and carvacrol methyl ether (5.6%). The antibacterial tests showed that the pathogenic bacteria *Staphylococcus aureus* and *Salmonella enterica* and the phytopathogenic bacteria *Erwinia rhapontici* and *Xanthomonas campestris* were the most susceptible to oregano oil, with the lowest concentrations of oil necessary to inhibit their bacterial growth. Moreover, oregano oil showed antibacterial activity against bacteria associated with food poisoning. In conclusion, *O. vulgare* from the arid Andean region possesses an important antibacterial activity with a high potential in the food industry and agriculture.

## 1. Introduction

There is a growing interest in assessing the antimicrobial and antioxidant properties of substances from natural sources that can potentially be used by the food and pharmaceutical industries. Essential oils from aromatic and medicinal plants have been known to possess biological activity [1,2]. Oregano (*Origanum vulgare*) is an aromatic plant with a wide distribution throughout the Mediterranean area and Asia [3]. Although oregano is an herb native to the European countries, it was also introduced to the Chilean territory more than 100 years ago [4]. In the arid Andean region, this plant is cultivated between 2800 and 3500 m above sea level. It produces a very aromatic spice with a specific quality or characteristics that are essentially due to the arid geographical environment [5,6,7]. This spice has long been used as a medicinal herb in ethnopharmacological preparations to treat various ailments such as respiratory disorders, dyspepsia, painful menstruation, rheumatoid arthritis, scrofulosis, and urinary tract disorders [8,9,10,11]. The high content of volatile essential oils is responsible for the antimicrobial activity, aroma, and flavor [12]. According to phytochemical studies on the essential oil composition of *Origanum vulgare* and other related species, there is a wide chemical diversity, with a considerable intraspecific qualitative and quantitative variation in constituents is found [13,14,15,16,17]. Some studies have also reported the potential of oregano essential oil to preserve food such as salmon and seaweed burgers [18], fish, and meat products [19]. Different studies on the isolation and characterization of *O. vulgare* essential oils from different world regions have been performed [20]. Nevertheless, there was no available information on the bioactive properties and characterization of *O. vulgare* essential oil cultivated in the arid Andean region. Therefore, this study aimed to describe the chemical composition of Andean *O. vulgare* essential oil and evaluate the antimicrobial and antioxidant activity of the oregano essential oil, as well as its potential as a food additive.

## 2. Results and Discussion

### 2.1. Physicochemical Properties

Table 1 shows the conditions, yields, and physical properties of the essential oil of oregano obtained by hydro-distillation. The density was 0.913 g/mL, and the refractive index was 1.4775; these values are similar to those reported previously for the same spice [21,22]. According to some research, the refractive index and density are proportionally related to the amount of phenols present [23]. The acidity index was 0.8345 mg of KOH/g of sample, equivalent to 0.419 g of oleic acid/100 g of oil.

### 2.2. Chemical Composition of the Essential Oil

**Figure 1:**
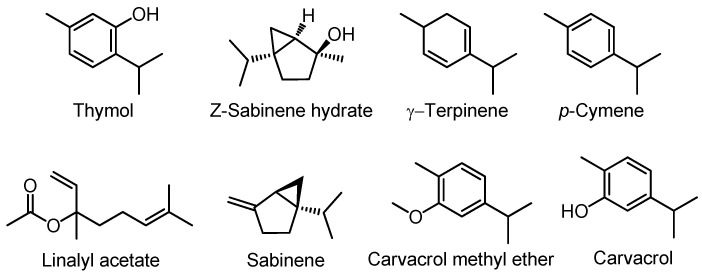
Chemical structures of the main compounds present in the essential oil of *O. vulgare* from Putre, Chile.

The *Origanum vulgare* essential oil composition was analyzed by GC-MS. The compound identification was carried out by comparing the relative retention times and the mass spectra of oil components with authentic samples and mass spectra from the data library. In total, fifty compounds were identified in *O. vulgare* essential oil, accounting for 99.2% of the whole composition, and they are mostly monoterpene hydrocarbons (38.7%), oxygenated monoterpenes (38.0%), and phenolic monoterpenes (19.0%). Within the monoterpene hydrocarbons, δ-terpinene (10.6%), *p*-cymene (8.6%), and sabinene (6.5%) were the major compounds detected, while *Z*-sabinene hydrate (13.4%), linalyl acetate (7.2%), and carvacrol methyl ether (5.6%) were the most abundant oxygenated monoterpenes detected (Figure 1, Table 2). Additionally, thymol (15.9%) represented the main fraction of *O. vulgare* essential oil. Carvacrol was also identified, but in a lower concentration (3.1%). The phytochemical contents of oregano oil from the arid region, especially the phenolic monoterpenes, curiously exhibited a great difference compared to what was reported by other authors [14,16,24,25].

In most cases, carvacrol constituted the major component (12.6–88.7%) of the oil, while the sum of the two phenolic monoterpenes (carvacrol and thymol) and their biosynthetic precursors *p*-cymene and γ-terpinene [27] represented approximately 75% of each essential oil [28,29]. Other compounds have also been reported as important essential oil components, such as caryophyllene, spathulenol, and germacrene-D [30,31]. However, the *Origanum* composition depends on the climate, altitude, time of recollection, and the stage of growth [32]. In this context, previous works on the phytochemical content of oregano oil grown in the northwestern Himalayas at 3200 m above sea level reported low values for carvacrol (1.1%) [33]. These results are in agreement with our observations on oregano grown at 3000 m above sea level. One of the arid Andean region’s main environmental characteristics is its high radiation levels, which would favor the concentration of some phenolic monoterpenes in the essential oil, as reported by Naghdi Badi [34].

On the other hand, a study in the semi-arid zones of North Africa shows that the essential oil from oregano grown in this region is mainly composed of thymol, *p*-cymene, γ-terpinene, and carvacrol [13]. It has also been reported that the concentration of *p*-cymene and γ-terpinene is high only in the respective poor oils of carvacrol and thymol, such as the essential oil of *Satureja thymbra* [29]. In short, we have made it clear that the biosynthesis of secondary metabolites of the essential oil of oregano grown in the arid Andean region studied is strongly affected by environmental factors. Agricultural practices also have a critical effect on the quantitative and qualitative characteristics of plant-derived metabolites.

### 2.3. Antioxidant Activity and Total Polyphenols Content

Because the antioxidant capacity cannot be fully described by a single method, for this study, the antioxidant capacities of *O. vulgare* essential oil were assayed with three different assays. Free radical scavenging capacities were measured using DPPH radical and ABTS radical cation. The values found are given in Table 3. The DPPH radical scavenging activity of the oregano oil from the arid region was very low (4750 ± 91.11 μmol Trolox/g); however, our result agrees with other authors’ observations on oregano oil ranging from 1509 μg/mL to 8900 μg/mL [24,30]. The value found for ABTS (1252.74 μmol Trolox/g) is higher than that reported by Sarikurkcu for the same species cultivated at 372 m above sea level [8]. Major components of the oregano oils may explain these differences in free radical scavenging capacities [16,35,36].

The reducing power of an essential oil can serve as a significant indicator of its potential antioxidant activity. For this reason, the reducing power of ferric ions and total polyphenols content were examined (Table 3). The ferric reducing antioxidant power (FRAP) of the essential oil in this study exhibited a weak reducing power compared to the values obtained in other works [8], but the total phenolic content (102.71 ± 3.87 mg GAE/g sample) was higher than those reported for the same species (4.1–17.7 mg GAE/g sample) [13,37,38] and slightly less than those reported for wild oregano populations from Sicily, Italy [39]. The lack of correlation between total polyphenol content and antioxidant capacity could be attributed to the fact that the Folin–Ciocalteu method reflects the presence of all reducing substances in a matrix, not just polyphenolics. Thus, the presence of non-phenolic substances cannot be ruled out. Therefore, interpretations of total phenolic content (TPC) results must consider these potential limitations. Despite these limitations, the TPC and antioxidant methods are widely used [40].

### 2.4. Antibacterial Activity

**Figure 2:**
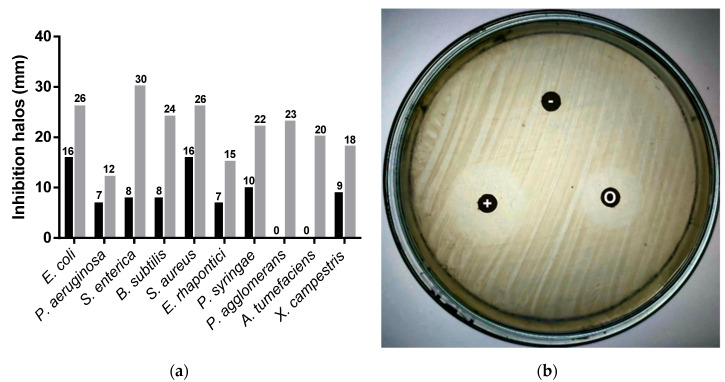
(**a**) Inhibitory activity (mm) of *O. vulgare* essential oil against pathogens and five phytopathogenic bacteria. (■) oregano; (■) kanamycin positive control. (**b**) Paper disk diffusion assay of oregano essential oil against *Escherichia coli*. Disk O: oregano oil; disk +: kanamycin positive control; disk −: ethanol negative control.

Figure 2 shows the inhibitory diameters for the oregano essential oil against five common foodborne pathogens and five phytopathogenic bacteria (Figure 2). This technique is recognized as a useful semi-quantitative method to determine the sensitivity of microorganisms to certain compounds. Disks impregnated in ethanol were used as a negative control. These disks were dried under the biosafety chamber’s flow to evaporate the ethanol and avoid its antimicrobial effect. The essential oil showed inhibitory activity against these pathogens, with diameters between 7 and 16 mm, classified as moderate/mild inhibitory activity compared to those reported by other authors [30,41,42]. As observed, almost all bacteria were sensitive to the inhibitory effect of oregano essential oil, except *Pantoea agglomerans* and *Agrobacterium tumefaciens*. It presented an inhibitory effect against *Pseudomonas aeruginosa*, contrary to what has been reported by other authors [31,42].

Table 4. shows the inhibitory and bactericidal effects of oregano oil, tested on five species of bacteria of clinical importance (*Escherichia coli*, *Pseudomonas aeruginosa*, *Salmonella enterica*, *Bacillus subtilis*, and *Staphylococcus aureus*) and five species of plant-pathogenic bacteria (*Erwinia rhapontici*, *Pseudomonas syringae*, *Pantoea agglomerans*, *Agrobacterium tumefaciens*, and *Xanthomonas campestris*). The different bacterial species tested showed varying susceptibility to the effects of the essential oil, which was expected since bacterial susceptibility to antimicrobial compounds is variable even at the strain level. This variation is due to specific differences in the composition of the different macromolecules and structures that these microorganisms possess. From Table 4, it can be observed that almost all microorganisms were susceptible to the action of oregano essential oil, with a variation in the MIC values from 0.04% *v/v* to 0.63% *v/v* and MBC values from 0.08% *v/v* to 1.25% *v/v*. *P. agglomerans* and *A. tumefaciens* showed resistance to the antimicrobial activity, and *P. aeruginosa* presented the highest resistance (0.63% *v/v* MIC and 1.25% *v/v* MBC). Kanamycin (50 µg) was used as a positive control of bacterial inhibition. *E. rhapontici* (0.04% *v/v* MIC and 0.08% *v/v* MBC) and *X. campestris* (0.08% *v/v* MIC and 0.08% *v/v* MBC) were the most susceptible phytopathogenic bacteria to oregano oil, with the lowest concentrations necessary to inhibit bacterial growth. These results could indicate a potential use of this essential oil in agriculture and agribusiness [43]. On the other hand, among the bacteria of clinical importance, *S. aureus* and *S. enterica* (0.08% *v/v* MIC and 0.08% *v/v* MBC) were the most susceptible to oregano oil. These results represent a promising strategy for controlling common foodborne pathogens.

The high sensitivity of all of the tested strains to the essential oil of *O. vulgare* is of particular interest. This antimicrobial effect could be attributed to an alteration in the lipidic components of the bacterial plasma membrane, resulting in the leakage of intracellular contents [44]. Furthermore, *O. vulgare* essential oil’s higher antimicrobial activity could be explained by the amount of phenolic oxygenated monoterpenes—especially thymol, *p*-cymene, and carvacrol, which were found in the ratio of 27.6% and they could show a synergistic effect causing membrane destabilization. Some studies ensure that the combination of carvacrol and thymol at low concentrations could be used as a reference to apply this combination in foods to control *S. aureus*, to maintain the organoleptic properties, and to extend the shelf-life of them [45]. Other studies have reported the synergic potential of oregano essential oil to preserve food, such as salmon and seaweed burgers [18], beef meatballs [46], fish and meat products [19]. In this context, the activity found in essential oil from the arid Andean region revealed its potential as a food additive to combat common foodborne pathogens.

## 3. Materials and Methods

### 3.1. Preparation of O. vulgare Essential Oil

Aerial parts of *Origanum vulgare* were collected cultivated in Socoroma (Putre, Chile) (18°15’4.67’’ S; 69°36’7.44’’ W, 2945 masl). The collection was carried out as described by Mechergui et al. [13], and Bisht et al. [33], with some modifications. Each collection consisted of 20 plants at the bloom stage in November 2019 from four different estates in the same village. The samples were selected to provide a homogenous group based on harvest, color, size, and freshness according to visual analysis. This sample was identified and deposited in the Herbarium from the Botany Department of Universidad de Concepción, Chile (voucher specimen 184934). The essential oil was obtained from dry plant material (50 g) by hydrodistillation for 60 min using a modified Clevenger system. Subsequently, the essential oil was dried through anhydrous sodium sulfate, and the yield was 5.3% (*v/d.w.)* of the plants collected in 2019. The essential oil was stored at −20 °C.

### 3.2. Chemicals

Phosphate buffer, trichloroacetic acid, ferric chloride, hydrochloric acid, ascorbic acid, 2,4,6-tris(2-pyridyl)-*s*-triazine (TPTZ), Folin–Ciocalteu reagent, gallic acid, 2,2-diphenyl-1-picrylhydrazyl (DPPH), potassium hexacyanoferrate(III), 6-hydroxy-2,5,7,8-tetramethylchroman-2-carboxylic acid (Trolox), 2,2′-azino-bis(3-ethylbenzothiazoline-6-sulfonic acid) (ABTS), and dimethylsulfoxide were from Sigma-Aldrich (Santiago, Chile); ferrous sulfate, sodium acetate, sodium carbonate, sodium persulfate, sodium sulfate anhydrous, and ethanol were from Merck (Santiago, Chile).

### 3.3. Gas Chromatography–Mass Spectrometry Analysis

GC-MS analysis was carried out as described by Teixeira et al., with some modifications [24]. The essential oil was analyzed using an Agilent 5975 gas chromatograph coupled to an Agilent 5973N mass-selective detector (Agilent Technologies, Palo Alto, CA., USA). It was fitted with an HP-5MS capillary column (30 m length × 0.32 mm internal diameter × 0.25 µm film thickness). The oven temperature was programmed at 45 °C for 1 min, raised to 250 °C at 5 °C min^−1^, and maintained at 250 °C for 5 min. Helium was used as carrier gas at 30 cm s^−1^, and the injection volume was 1 µL. The identity of each compound was assigned by comparing their retention index relative to a standard mixture of *n*-alkanes, as well as by comparison with the mass spectra characteristic features obtained from the Wiley data bank (Wiley 7N Edition [Agilent Part No. G1035B]: Wiley Registry of Mass Spectral Data, 7th Edition), whenever possible, co-injections with authentic samples.

### 3.4. Determination of Physicochemical Characteristics

Physicochemical characteristics were evaluated as described by Tellez-Monzón et al. [21], with some modifications. The fresh essential oil’s specific density was measured using a 10 mL pycnometer at 20 °C. The weight of the empty pycnometer was recorded. The pycnometer was filled with the essential oil and weighed again. The experiment was carried out in triplicate. The density of the essential oils at room temperature was calculated using the formula:

(1) $$\mathrm{Density}\;=\;\frac{mass}{volume}$$

The refractive index of the essential oils at room temperature was measured using an ABBE REF-1 refractometer (PCE Instruments, Meschede, Germany). The acidity index was determined through a potentiometric titration with a 0.02 N NaOH solution and expressed as a percentage of oleic acid.

### 3.5. Determination of Total Phenols (Folin-Ciocalteu)

Total polyphenol content was determined using the Folin-Ciocalteu method, with some modifications [38]. From the 50 g/L solution, one was prepared at 0.5 g/L, where an aliquot of the solution (1000 μL) was mixed with 500 μL of Folin-Ciocalteu reagent (50% *v/v*) and 5000 μL of ethanol at 80% *v/v*. After 5 min of reaction, 250 µL of 5% *w/v* Na_2_CO_3_ was added and brought to a final volume of 8000 µL with 80% *v/v* ethanol. This was then allowed to incubate for 30 min at room temperature. Each sample was measured at 760 nm and compared to a calibration curve using gallic acid as the standard.

### 3.6. Antioxidant Activity Assays

#### 3.6.1. Ferric Reducing Antioxidant Power (FRAP)

The determination of the ferric reducing antioxidant power or the ferric reducing capacity (FRAP test) was carried out as described by Parra et al. [47], with some modifications. The FRAP reagent was prepared by mixing 25 mL of acetate buffer (300 mmol/L, pH 3.6), 2.5 mL of TPTZ solution (10 mmol/L) in hydrochloric acid (40 mmol/L), and 2.5 mL of FeCl_3_·6H_2_O (20 mmol/L). For measurement, 25 µL of diluted extract (0.5 g/L) was mixed with 175 µL of FRAP reagent in a 96-well microplate. The mixture was incubated for 40 min at 25 °C, and then absorption was recorded at 593 nm in a Synergy ™ HTX multimodal microplate reader (BioTek Instruments, Inc., Winooski, VT, USA). To quantify each solution’s antioxidant capacity, Trolox was used as standard and ethanol 80% *v/v* as blank.

#### 3.6.2. Free Radical Scavenging (DPPH)

The radical DPPH methodology was used with some modifications [38]. In an analytical balance, 0.0197 g of material was ground and brought to a final volume of 50 mL in 80% ethanol. A 100 µL aliquot of 1 mM DPPH was then mixed with 100 µL of gallic acid standard (15–120 ppm) and solution of essential oil from 2 g/L to 50 g/L. The absorbance was fixed at 517 nm, with shaking for 5 min and incubation for 30 min at 36 °C. The percentage of radical inhibition DPPH was calculated according to the following formula:

(2) $$\%\;=\;\frac{\left({Abs}_{\mathrm{control}}\;-\;{Abs}_{\mathrm{sample}}\right)}{{Abs}_{\mathrm{control}}}\;\times \;100$$

Subsequently, a curve of percent inhibitory activity of DPPH versus concentration of the extract was drawn, and the IC_50_ value was calculated. 

#### 3.6.3. ABTS Method

The ABTS assay was performed by bleaching the cationic radical ABTS^•+^ as described by Soto et al. [48]. For the preparation of the radical ABTS^•+^, 2.5 mL of the 7 mM ABTS solution was mixed with 2.5 mL of 2.45 μM sodium persulfate for 12 h in the dark at 4 °C. Then, the resulting solution was diluted with absolute ethanol until an initial absorbance of approximately 0.70 ± 0.03 was obtained at 734 nm. The radical discoloration was initiated by adding 50 µL of the extract to 150 µL of the ABTS^•+^ solution. After 15 min of incubation at 25 °C, the absorbance was measured at 734 nm and compared with a calibration curve using Trolox as standard and ethanol as blank. Results were expressed as millimoles of Trolox equivalents per gram of dry sample (mmol TE/g).

### 3.7. Antibacterial Activity

#### 3.7.1. Strain and Growth Conditions

Oregano oil was used to determine antibacterial activity against the human pathogenic bacteria *Escherichia coli* (ATCC 23716), *Pseudomonas aeruginosa* (ATCC 19429), *Salmonella enterica* (ATCC 13311), *Bacillus subtilis* (ATCC 6051), and *Staphylococcus aureus* (ATCC 29737); and the phytopathogenic bacteria *Erwinia rhapontici* (MK883065), *Pseudomonas syringae* (MF547632), *Pantoea agglomerans* (MK883087), *Agrobacterium tumefaciens* (ATCC 19358), and *Xanthomonas campestris* (MH885473). Bacteria were inoculated into nutrient broth containing 5.0 g/L peptone and 3.0 g/L meat extract and incubated at 25 °C (plant pathogens) or 35 °C (human pathogens) for 18 h at 150 rpm using an incubator with orbital shaking LOM-80 (MRC Lab, London, UK).

#### 3.7.2. Paper Disk Diffusion Method

The antibacterial activity of oregano oil was determined by the method described by Klančnik et al., with modifications [49]. A stock solution of 10% (*v/v*) oregano oil in ethanol was prepared. An aliquot of 15 μL of the stock solution was impregnated on 5 mm sterile cellulose filter paper disks. The same procedure was repeated with disks impregnated only with ethanol, which were used as a negative control. The impregnated disks were dried under the biosafety chamber flow to evaporate ethanol and avoid its antimicrobial effect. Disks containing 50 μg kanamycin were used as a positive control of bacterial inhibition. Fresh inocula of each bacterial species were prepared as previously described, diluted to 0.5 of McFarland standard, and inoculated uniformly on plates containing nutrient broth supplemented with 12 g/L of agar. The dried and impregnated disks were arranged equidistant from each other. The plates were incubated for 24 h at 25 °C or 35 °C, as appropriate. Bacterial growth inhibition diameter was recorded after the incubation step, which is observed as a transparent halo without growth around each disk. The tests were carried out in triplicate.

#### 3.7.3. Minimum Inhibitory Concentration (MIC)

The minimum concentration of oregano oil necessary to inhibit bacterial growth was carried out using the method described by Andrews [50]. The final oregano oil concentrations used were 0 to 10% (*v/v*). The final working volume for each concentration was 200 µL. Each dilution was inoculated with the different bacteria to be tested in 96-well plates at 25 °C or 35 °C, as appropriate. As controls, nutrient broth without compound inoculated with each bacterium (growth control), and nutrient broth with 0–10% (*v/v*) of oregano oil without bacterial inoculation (negative control of growth and sterility of the compound) were used. An assay using 0–90% ethanol was performed to estimate the ethanol MIC of each bacterium. Concentrations higher than 20% ethanol were shown to be inhibitory for all bacteria. For this reason, a 40% (*v/v*) oregano oil was prepared as a stock solution to avoid the inhibitory effect of ethanol during MIC assays. After 24 h of incubation, we determined the lowest concentration of compound at which no bacterial growth was observed.

#### 3.7.4. Minimum Bactericidal Concentration (MBC)

The bactericidal capacity of oregano oil was determined from the last three wells that showed no bacterial growth in the MIC assay, as described by Taylor et al. [51]. For this, 100 µL of these cultures were taken and plated in nutrient broth plates supplemented with agar. As a growth control, a culture that did show microbial growth in the MIC test was used. The plates were incubated for 24 h at the corresponding temperature, after which the concentration of oregano oil where no growth was observed (MBC) was determined for each microorganism.

## 4. Conclusions

This work determined the chemical composition of the essential oil of *Origanum vulgare* grown in the arid Andean region (Putre, Chile). The main components of the essential oregano oil were thymol (15.9%), Z-sabinene hydrate (13.4%), γ-terpinene (10.6%), and *p*-cymene (8.6%). The antioxidant activity found was not related to its chemical composition. We also investigated the use of oregano essential oil as an antimicrobial agent against several bacteria. In this context, *O. vulgare* oil is a natural source of antibacterial compounds and has strong potential as a food additive or for agricultural applications. Nonetheless, safety and toxicity issues of this essential oil still need to be evaluated.

In summary, this study expands the knowledge of *O. vulgare* species cultivated under environmental conditions typical of the Atacama Desert’s Andean region. This knowledge could help to conduct more research on *O. vulgare* and strengthen its potential use as a natural source of bioactive compounds that could drive the development of local, regional, and international trade as a vehicle for rural economic growth in this region.

## Figures and Tables

**Table 1:** Physicochemical characteristics of *Origanum vulgare* essential oil from Putre, Chile.

Parameters	*Origanum vulgare* L.
Distillation time (min)	60
Yield (%)	1.89
Specific density 20 °C (g/mL)	0.913
Refractive index	1.4775
Acidity index (mg KOH/g sample)	0.8345

**Table 2:** Chemical composition of volatiles in the *Origanum vulgare* essential oil from Putre, Chile.

N°	Compounds	RI ^1^	Ref RI ^2^	%	Identification
1	α-Thujene	927	924	1.2	a
2	α-Pinene	936	932	0.7	a,b
3	Camphene	951	946	tr	a,b
4	Sabinene	971	974	6.5	a
5	β-Pinene	978	976	0.3	a,b
6	1-Octen-3-ol	978	979	0.2	a,b
7	Eucalyptol	988	988	tr	a
8	β-Myrcene	991	988	1.9	a,b
9	α-Phellandrene	994	1001	0.5	a
10	3-Octanol	993	991	tr	a
11	δ-3−Carene	1008	1008	tr	a
12	α-Terpinene	1020	1015	3.5	a
13	*Z*-β-Ocimene	1027	1032	0.9	a
14	*p*-Cymene	1028	1024	8.6	a,b
15	Limonene	1033	1027	0.9	a,b
16	*E*-β-Ocimene	1047	1044	0.2	a
17	α-Terpinolene	1053	1053	0.8	a
18	γ-Terpinene	1062	1058	10.6	a,b
19	*Z*-Sabinene hydrate	1073	1066	13.4	a
20	*Z*-Linalool oxide	1075	1067	tr	a
21	*E*-Linalool oxide	1082	1084	tr	a
22	*E*-Sabinene hydrate	1105	1098	2.0	a
23	β-Thujone	1114	1112	2.2	a
24	*E-p*-2-Menthen-1-ol	1124	1129	0.4	a
25	*endo*-Borneol	1160	1165	0.2	a
26	*p*-Cymen-8-ol	1180	1179	0.1	a
27	4-Terpineol	1179	1177	4.4	a
28	*p*-Cymen-7-ol	1181	1181	tr	a
29	α-Terpineol	1186	1186	1.8	a
30	*E*-Piperitol	1193	1207	0.3	a
31	*Z*-Piperitol	1207	1195	0.3	a
32	Nerol	1229	1227	tr	a
33	Thymol methyl ether	1237	1232	2.3	a
34	Carvacrol methyl ether	1245	1241	5.6	a
35	Geraniol	1250	1249	tr	a
36	Linalyl acetate	1256	1257	7.2	a
37	Bornyl acetate	1289	1284	tr	a
38	Thymol	1293	1289	15.9	a,b
39	Carvacrol	1300	1298	3.1	a,b
40	Eugenol	1357	1356	tr	a
41	Geranyl acetate	1380	1379	0.1	a
42	β-Caryophyllene	1420	1418	1.2	a,b
43	Aromadendrene	1442	1439	tr	a
44	α-Humulene	1454	1452	0.4	a
45	Bicyclogermacrene	1500	1494	1.5	a
46	δ-Cadinene	1524	1522	tr	a
47	Ledene	1552	1552	tr	a
48	Spathulenol	1573	1577	0.1	a,b
49	Caryophyllene oxide	1581	1582	0.1	a,b
50	Isospathulenol	1704	1704	tr	a
	Monoterpene hydrocarbons			38.7	
	Oxygenated monoterpenes			38.0	
	Phenolic monoterpenes			19.0	
	Sesquiterpene hydrocarbons			3.1	
	Oxygenated sesquiterpenes			0.2	
	Others			0.2	
	Total identified			99.2	

^a^ Comparison of mass spectra with MS libraries and retention times. ^b^ Comparison with authentic compounds. tr: concentration lower than 0.05%. Compounds are listed in order of their elution on the HP-5MS column. ^1^ Retention index on the HP-5MS column relative to C_8_–C_24_ *n*-alkanes. ^2^ Retention index from the literature [26].

**Table 3:** Antioxidant activity of *Origanum vulgare* essential oil from Putre, Chile.

Species	DPPH ^a^	ABTS ^b^	FRAP ^b^	TPC ^c^
*O. vulgare*	4750 ± 91.11	1252.74 ± 47.10	270.53 ± 3.52	102.71± 3.87
Quercetin *	6.99 ± 0.02	-	-	-
Trolox *	20.99 ± 1.24	-	-	-

^a^ Antiradical DPPH activities are expressed as IC_50_ in µg/mL for sample and compounds; ^b^ Expressed as μmol Trolox/g sample; ^c^ Total phenolic content (TPC) expressed as mg GAE/g sample; * Used as standard antioxidants.

**Table 4:** Antibacterial activity of *Origanum vulgare* essential oil from Putre, Chile.

Bacteria	MIC ^1^ (%)	MBC ^2^ (%)
**Pathogenic**		
*Escherichia coli* (ATCC 23716)	0.16	0.16
*Pseudomonas aeruginosa* (ATCC 19429)	0.63	1.25
*Salmonella enterica* (ATCC 13311)	0.08	0.08
*Bacillus subtilis* (ATCC 6051)	0.08	0.32
*Staphylococcus aureus* (ATCC 29737)	0.08	0.08
**Phytopathogenic**		
*Erwinia rhapontici* (MK883065)	0.04	0.08
*Pseudomonas syringae* (MF547632)	0.16	0.32
*Pantoea agglomerans* (MK883087)	ND	ND
*Agrobacterium tumefaciens* (ATCC 19358)	ND	ND
*Xanthomonas campestris* (MH885473)	0.08	0.08

^1^ Minimum inhibitory concentration. ^2^ Minimum bactericidal concentration. ND: inhibition not detected. ATCC: American Type Culture Collection (USA). MK883065, MF547632, MK883087, and MH885473 are the accession numbers of the respective bacteria in GenBank.
